# Supplementary material for: Control of osteocyte dendrite formation by Sp7 and its target gene osteocrin
Source: Nat Commun. 2021 Nov 1;12:6271. doi: 10.1038/s41467-021-26571-7 (PMC8560803; doi:10.1038/s41467-021-26571-7)
Supplement: Supplementary file 1 — Supplementary Information [file 41467_2021_26571_MOESM1_ESM.pdf]

SUPPLEMENTARY INFORMATION

Control of osteocyte dendrite formation by Sp7 and its target gene osteocrin

Jialiang S. Wang[1], Tushar Kamath[2.3], Courtney M. Mazur[1], Fatemeh Mirzamohammadi[1,4], Daniel Rotter[1,5], Hironori Hojo[6], Christian D. Castro[1], Nicha Tokavanich[1], Rushi Patel[1], Nicolas Govea[1,7], Tetsuya Enishi[1,8], Yunshu Wu[1,9], Janaina da Silva Martins[1], Michael Bruce[1], Daniel J. Brooks[1,10], Mary L. Bouxsein[1,10], Danielle Tokarz[11,12], Charles P. Lin[11], Abdul Abdul[2,3], Evan Z. Macosko[2,3,13], Melissa Fiscaletti[14], Craig F. Munns[15,16], Pearl Ryder[2,17], Maria Kost-Alimova[2,18], Patrick Byrne[2,18], Beth Cimini[2,17], Makoto Fujiwara[19], Henry M. Kronenberg[1], Marc N. Wein[1,2,20]

[1]Endocrine Unit, Massachusetts General Hospital, Harvard Medical School, Boston, MA
[2]Broad Institute of Harvard and MIT, Cambridge, MA
[3]Stanley Center for Psychiatric Research, Broad Institute of Harvard and MIT, Cambridge, MA
[4]Department of Plastic and Reconstructive Surgery, Wright State University, Dayton, OH
[5]University of Applied Sciences Technikum Wien, Vienna, Austria
[6]Center for Disease Biology and Integrative Medicine, The University of Tokyo Graduate School of Medicine, 7-3-1 Hongo, Bunkyo-ku, Tokyo 113-8656, Japan
[7]Department of Anesthesiology, Weill Cornell Medical School, New York, NY
[8]Department of Orthopedic Surgery, Tokushima Municipal Hospital, Tokushima, Japan
[9]State Key Laboratory of Oral Diseases, National Clinical Research Center for Oral Diseases, West China Hospital of Stomatology, Sichuan University, Chengdu, China
[10]Center for Advanced Orthopedic Studies, Department of Orthopedic Surgery, Beth Israel Deaconess Medical Center, Harvard Medical School, Boston, Massachusetts, USA.
[11]Advanced Microscopy Program, Center for Systems Biology and Wellman Center for Photomedicine, Massachusetts General Hospital, Harvard Medical School, Boston, MA
[12]Department of Chemistry, Saint Mary's University, Halifax, Canada
[13]Department of Psychiatry, Massachusetts General Hospital, Harvard Medical School, Boston, MA
[14]Pediatric Department, Sainte-Justine University Hospital Centre, Montreal, Canada
[15]Institute of Endocrinology and Diabetes, The Children's Hospital at Westmead, Sydney, NSW, Australia
[16]Discipline of Paediatrics & Child Health, University of Sydney, Sydney, 2006, Australia
[17]Broad Institute of Harvard and MIT, Imaging Platform, Cambridge, MA
[18]Broad Institute of Harvard and MIT, Center for the Development of Therapeutics, Cambridge, MA
[19]Department of Pediatrics, Osaka University Graduate School of Medicine, Osaka, Japan
[20]Harvard Stem Cell Institute, Cambridge, MA

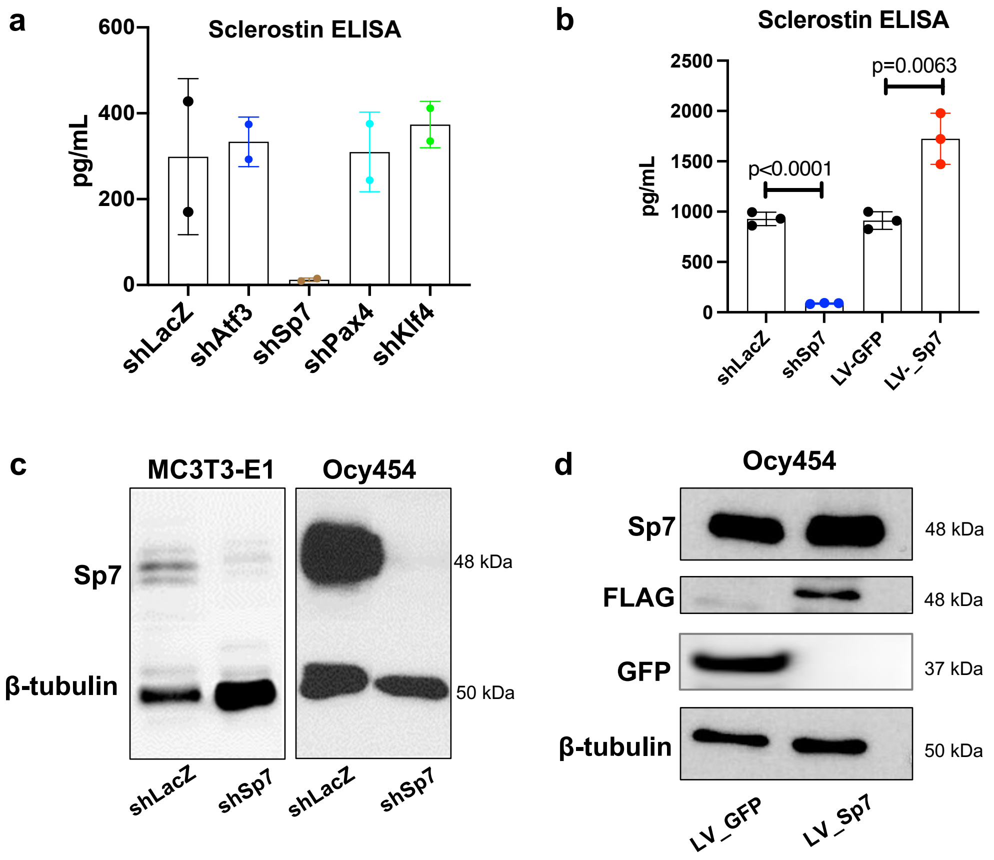

Fig. S1: (**a**) Ocy454 cells were infected with shRNA-expressing lentiviruses targeting the indicated gene followed by puromycin selection. Cells were grown at 37ºC for 14 days, then sclerostin ELISA was performed on 72h conditioned medium. Only *Sp7* knockdown reduced sclerostin level. Statistical analysis was not performed as biologic duplicates were collected. (**b**) Ocy454 cells were subjected to *Sp7* knockdown (left) or *Sp7* overexpression (right) followed by sclerostin ELISA. In panels (a and b), each data point represents a biologically independent sample. Data are presented as mean values +/- SD. Two-tailed students t-test was used to assess differences between Sp7 knockdown (versus control) and over-expression (versus control). Exact p values are indicated in the graph. (**c**) MC3T3-E1 and Ocy454 cells were infected with the shRNA-expressing lentiviruses followed by immunoblotting as indicated. shSp7 infection leads to near complete loss of Sp7 protein. (**d**) Ocy454 cells were infected with control (LV-GFP) or FLAG-Sp7 expressing lentiviruses, followed by immunoblotting as indicated. Representative immunoblots from two independent experiments are shown.

a

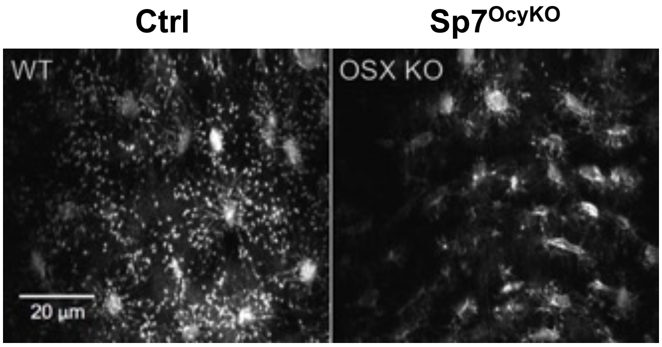

Fig. S2: (**a**) Representative *in vivo* third harmonic generation images from control and *Sp7*^{OcyKO} mice, revealing reduced punctate signal representing osteocyte dendrites. See also Fig. 1j, n, and o. Punctate structures likely represent filaments perpendicular to the imaging plane. However, the possibility remains that these punctate structures in THG imaging may also be fluid-filled extracellular vesicles (exosomes) as described by Qin and Dallas (*Curr Osteoporos Rep*, 2019).

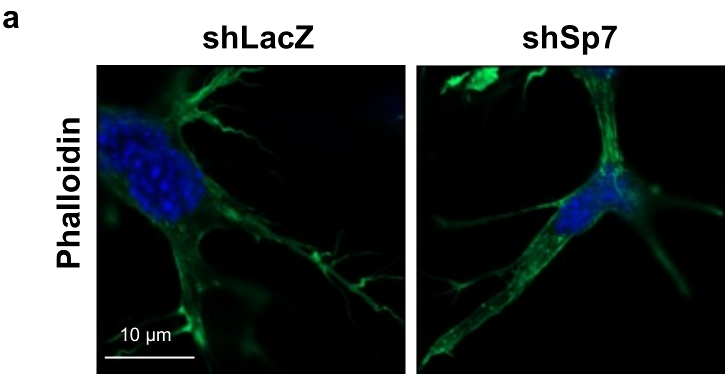

Fig. S3: (**a**) Ocy454 cells were infected with control or *Sp7* knockdown lentiviruses followed by growth in 3D culture. Similar to results seen in MC3T3-E1 cells (Fig. 1j), *Sp7* knockdown causes defective formation of phalloidin-positive long filaments in this cell type. Scale bar represents 10 µm.

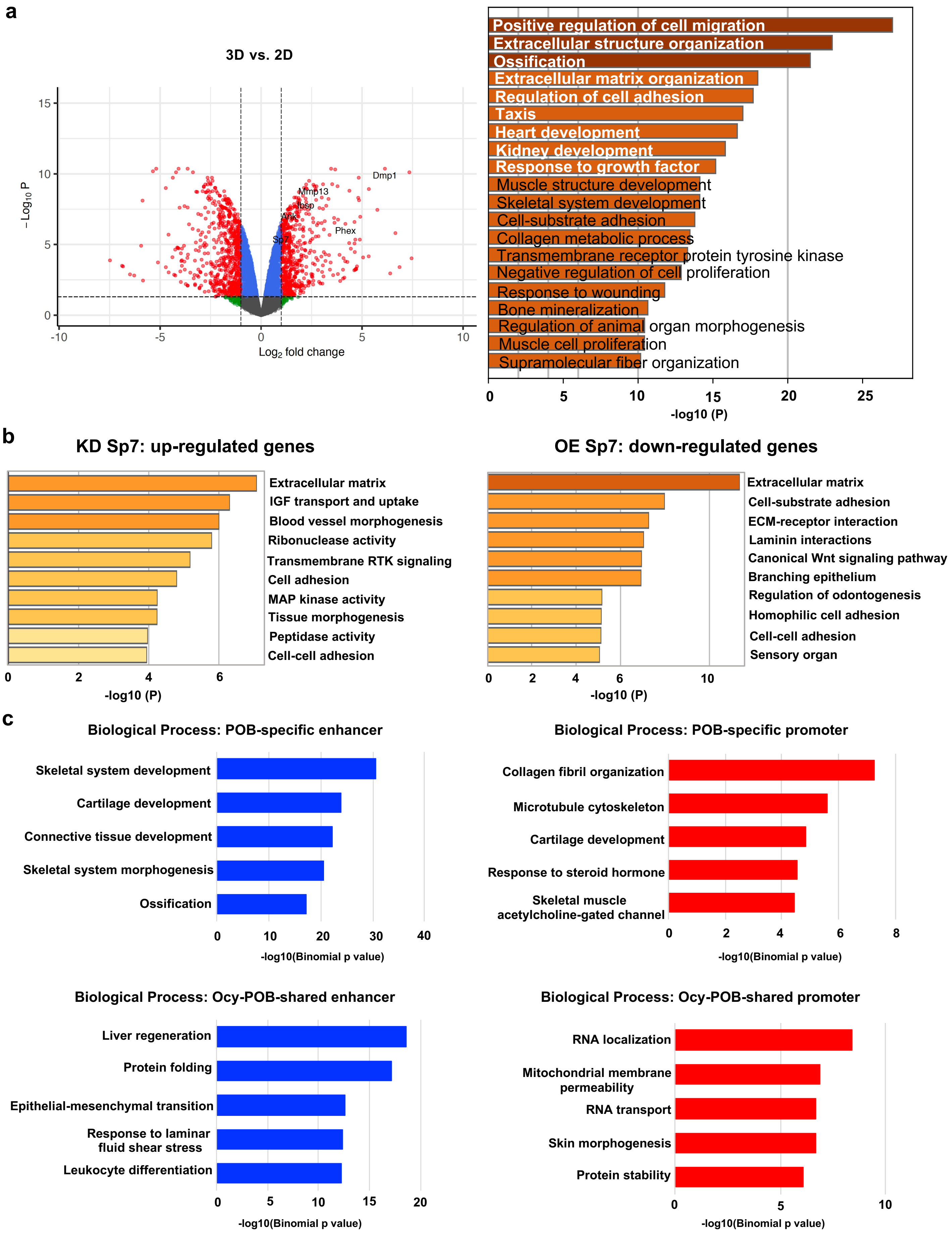
a
3D vs. 2D
−Log10 P
Log2 fold change
Dmp1
Mmp13
Ibsp
Ank
Sp7
Phex
Positive regulation of cell migration
Extracellular structure organization
Ossification
Extracellular matrix organization
Regulation of cell adhesion
Taxis
Heart development
Kidney development
Response to growth factor
Muscle structure development
Skeletal system development
Cell-substrate adhesion
Collagen metabolic process
Transmembrane receptor protein tyrosine kinase
Negative regulation of cell proliferation
Response to wounding
Bone mineralization
Regulation of animal organ morphogenesis
Muscle cell proliferation
Supramolecular fiber organization
-log10 (P)
b
KD Sp7: up-regulated genes
Extracellular matrix
IGF transport and uptake
Blood vessel morphogenesis
Ribonuclease activity
Transmembrane RTK signaling
Cell adhesion
MAP kinase activity
Tissue morphogenesis
Peptidase activity
Cell-cell adhesion
-log10 (P)
OE Sp7: down-regulated genes
Extracellular matrix
Cell-substrate adhesion
ECM-receptor interaction
Laminin interactions
Canonical Wnt signaling pathway
Branching epithelium
Regulation of odontogenesis
Homophilic cell adhesion
Cell-cell adhesion
Sensory organ
-log10 (P)
c
Biological Process: POB-specific enhancer
Skeletal system development
Cartilage development
Connective tissue development
Skeletal system morphogenesis
Ossification
-log10(Binomial p value)
Biological Process: POB-specific promoter
Collagen fibril organization
Microtubule cytoskeleton
Cartilage development
Response to steroid hormone
Skeletal muscle acetylcholine-gated channel
-log10(Binomial p value)
Biological Process: Ocy-POB-shared enhancer
Liver regeneration
Protein folding
Epithelial-mesenchymal transition
Response to laminar fluid shear stress
Leukocyte differentiation
-log10(Binomial p value)
Biological Process: Ocy-POB-shared promoter
RNA localization
Mitochondrial membrane permeability
RNA transport
Skin morphogenesis
Protein stability
-log10(Binomial p value)

Fig. S4: (**a**) Left: volcano plot from bulk RNA-seq data of MC3T3-E1 cells infected with shLacZ. Cells were grown in either 2D or 3D culture. Differentially expressed genes are defined as log2 FC >1 or <-1 with adjusted p value (FDR) < 0.05. Several osteocyte markers are highlighted: *Sp7*, *Dmp1, Phex, Ank*; Right: gene ontology analysis of differentially expressed genes in 3D culture reveals enrichment in several terms associated with cell migration, ossification and supramolecular fiber organization. (**b**) Gene ontology analysis of genes up-regulated in response to *Sp7* knockdown (KD, left) and down-regulated in response to *Sp7* overexpression (OE, right). (**c**) Top: functional analysis of genes bound by Sp7 only in osteoblasts (POB) at the enhancer (left) and promoter (right) region. Bottom: functional analysis of genes bound by Sp7 in both osteoblasts and Ocy454 cells at the enhancer (left) and promoter (right) regions. Fisher's exact test was used with multiple hypothesis adjustment for gene ontology analysis.

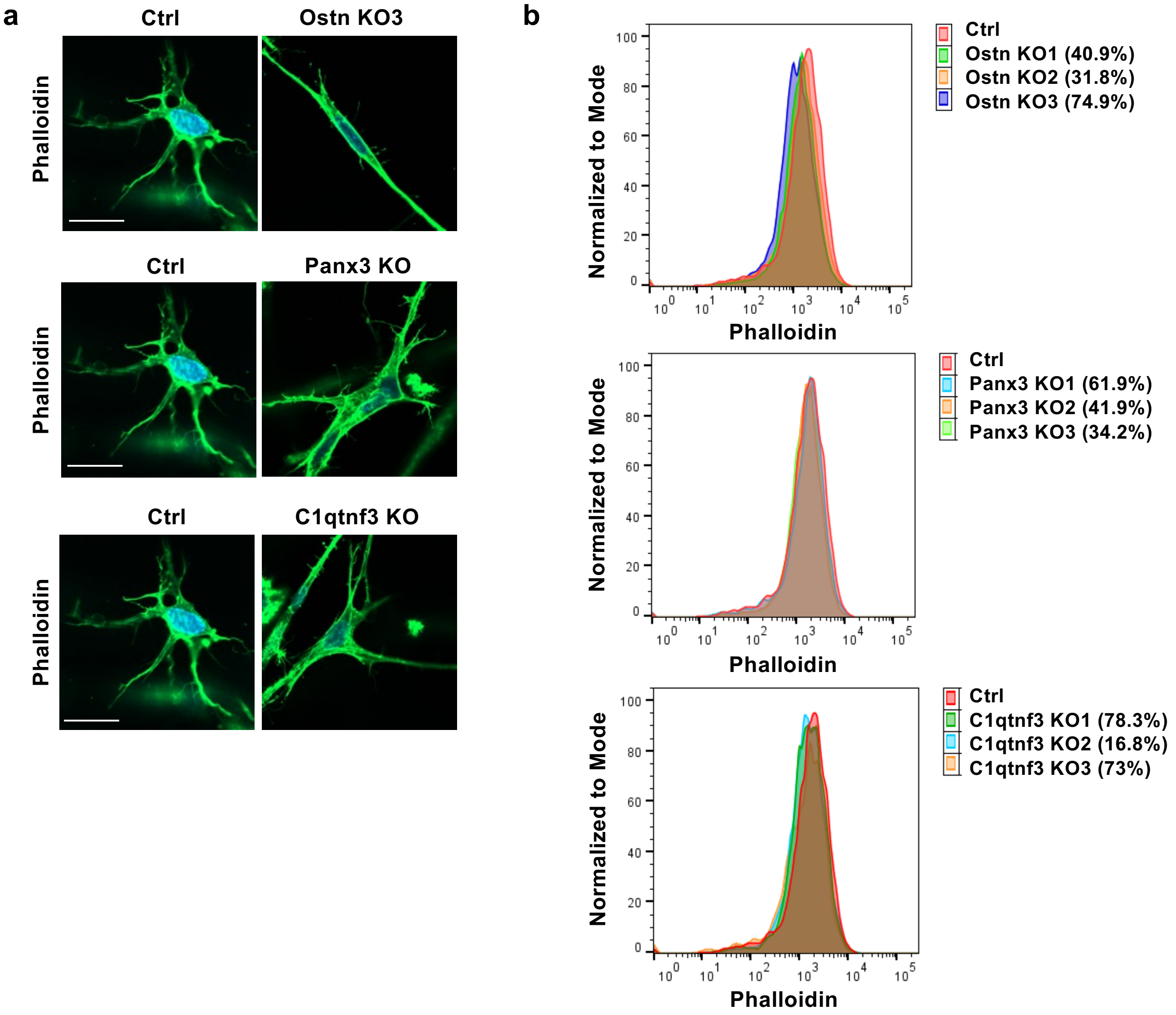

Fig. S5: Ocy454 cells were subjected to CRISPR/Cas9-mediated deletion of the indicated gene, followed by growth in 3D culture conditions and phalloidin staining (**a**) or flow cytometry (**b**). Representative images are shown from three independent experiments. Only *Ostn* CRISPR deletion led to defects in F-actin content. The percentage of gene editing efficiency as determined by TIDE is shown on the right for each sgRNA in panel (**b**). Scale bars represent 10 μm.

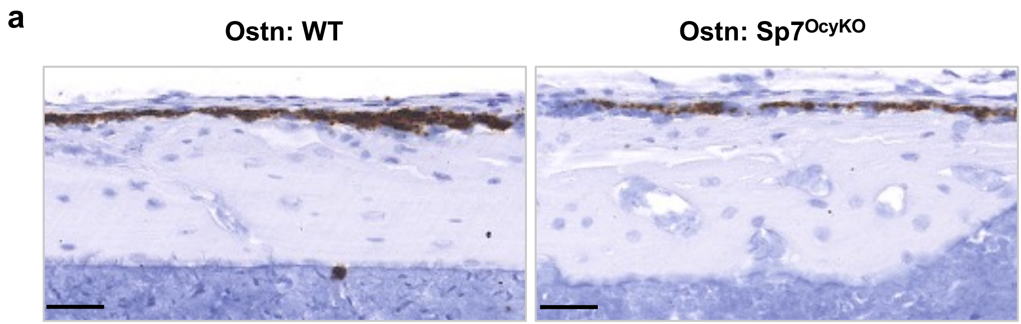

Fig. S6: (**a**) RNA *in situ* hybridization of *Ostn* in 6-week-old WT (left) and Sp7^{OcyKO} (right) mouse tibia. Scale bar represents 50 µm. Representative images are shown from three independent staining experiments.

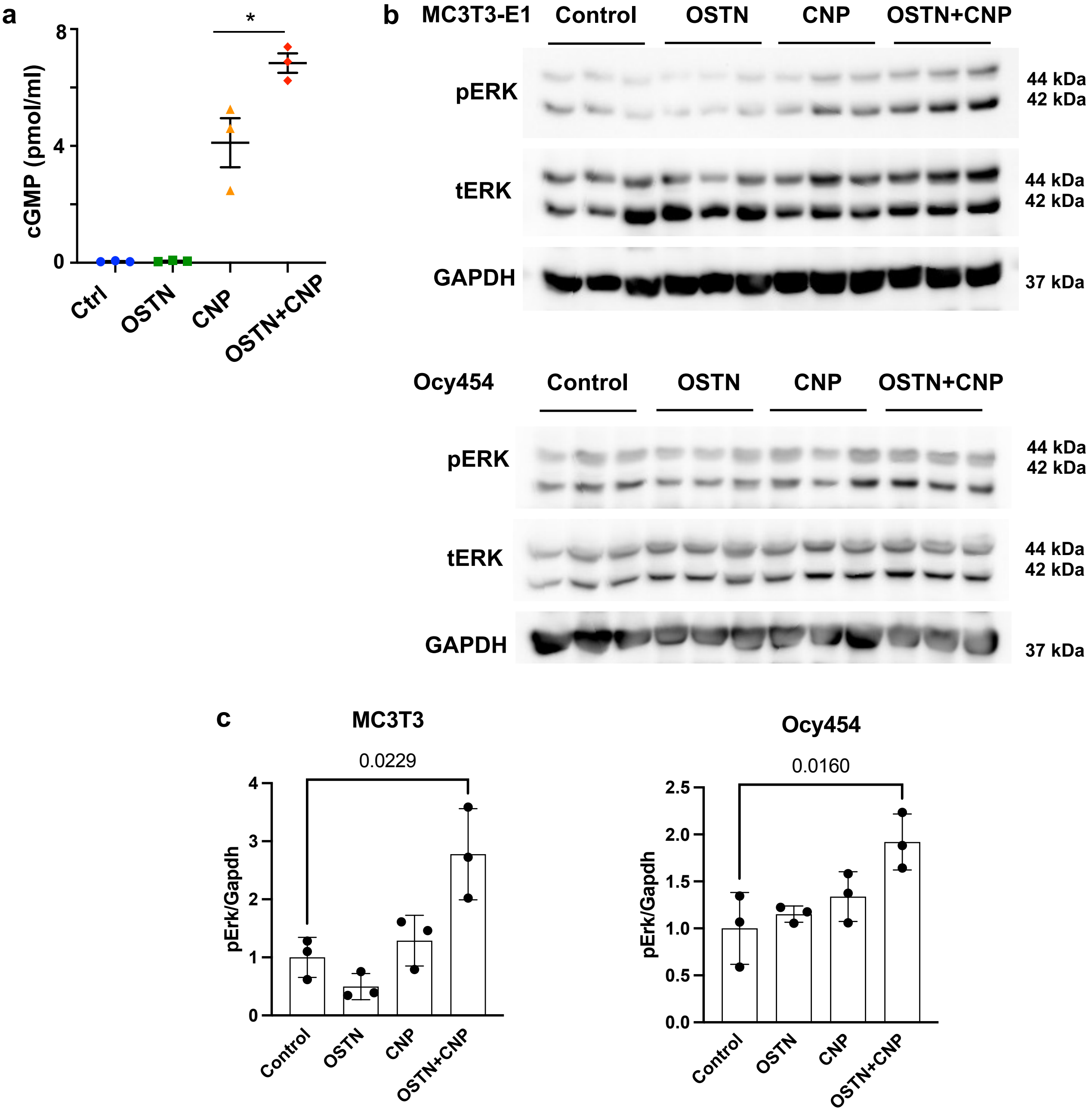

Fig. S7: (**a**) cGMP levels in Ocy454 cells were measured by ELISA. Cells were incubated for 30 minutes under control conditions (HBSS) or with 50 nM CNP, or 500 nM OSTN, or 50 nM CNP + 500 nM OSTN. Data are representative of 3 independent experiments. Ordinary one-way ANOVA was performed followed by Tukey's multiple comparisons test. *, p=0.0118. (**b**) Western blot of pERK, tERK and GAPDH. MC3T3-E1 (top) and Ocy454 (bottom) cells were treated with 100 nM CNP, or 500nM OSTN, or 100 nM CNP + 500 nM OSTN. Representative immunoblots are shown from three independent experiments. (**c**) Densitometry was performed to quantify immunoblotting results from panel (**b**). Graphs show mean +/- SD, Two-way ANOVA was performed followed by pairwise comparison of all groups versus control. Each data point represents densitometry results from an independent biologic replicate.

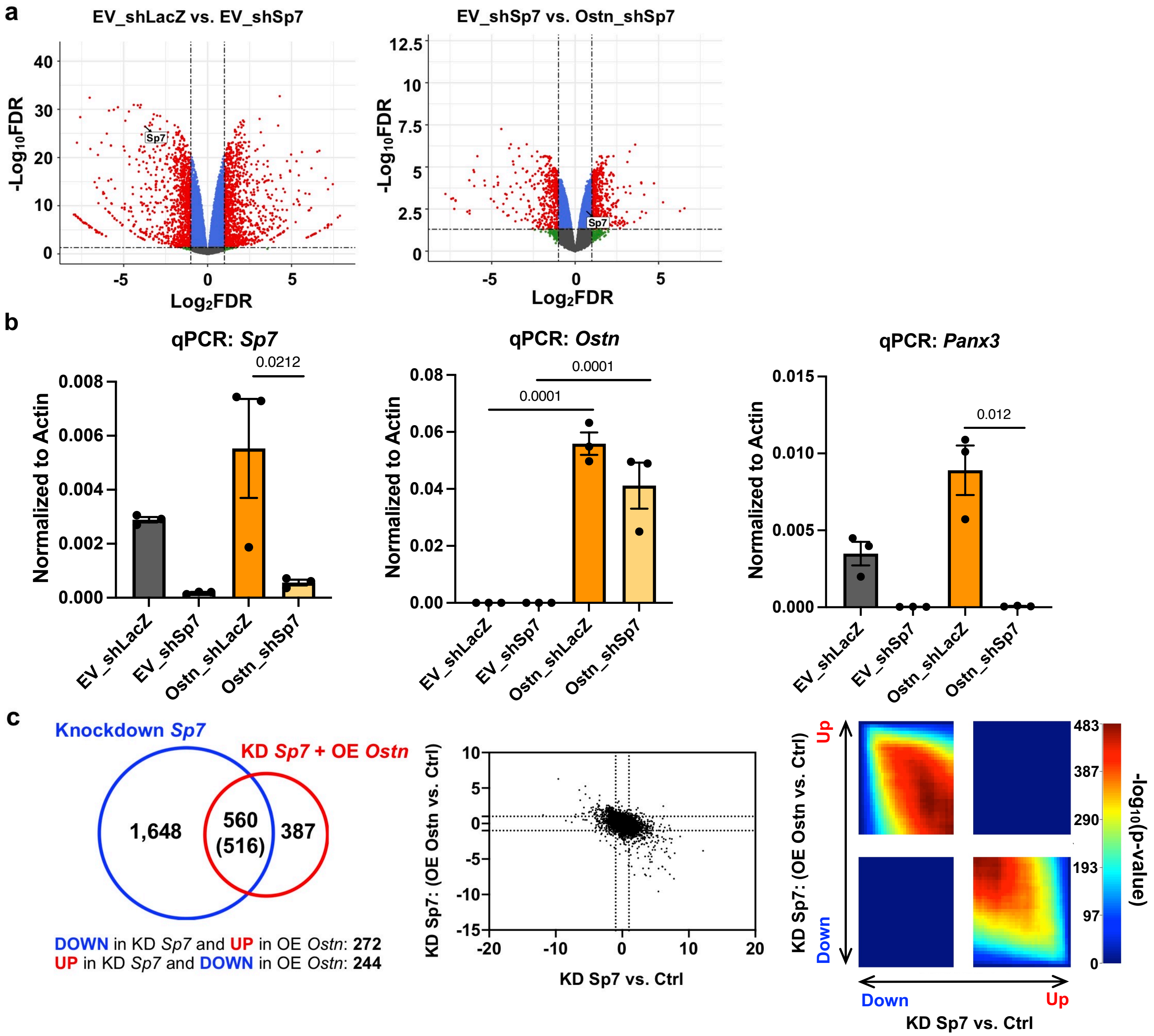

Fig. S8: (**a**) Left: volcano plot from bulk RNA-seq data of MC3T3-E1 cells infected with shSp7 versus control (shLacZ). Right: volcano plot from bulk RNA-seq data of MC3T3-E1 cells infected with shSp7 then subjected to control (EV, empty vector) or *osteocrin* (*Ostn*) over-expression. Differentially expressed genes are defined as log2 FC >1 or <-1 with adjusted p value (FDR) < 0.05. (**b**) Cells as in (**a**) were analyzed by RT-qPCR for the indicated genes, demonstrating expected degrees of *Sp7* knockdown and *Ostn* over-expression. *Panx3* is a Sp7-dependent gene whose expression is not rescued by Ostn. Each data point represents a biologically independent sample. Data are presented as mean values +/- SEM. 2-way ANOVA with Tukey's multiple comparison test was performed, exact p values are shown on the graphs. (**c**) Left, RNA-seq was performed in control (shLacZ) versus *Sp7* knockdown MC3T3-E1 cells (blue circle), and *Sp7* knockdown cells rescued with control (LV-empty vector) or osteocrin cDNA (red circle). The intersection of differentially-expressed genes in these two comparisons revealed 560 common transcripts, the majority (516) of which were discordantly regulated by the two perturbation. Middle, scatterplot analysis between the effects of *Sp7* knockdown and *osteocrin* expression in *Sp7* knockdown cells. Right: RRHO2 visualization revealing statistically significant groups of genes counter-regulated by *Sp7* knockdown and *Ostn* overexpression.

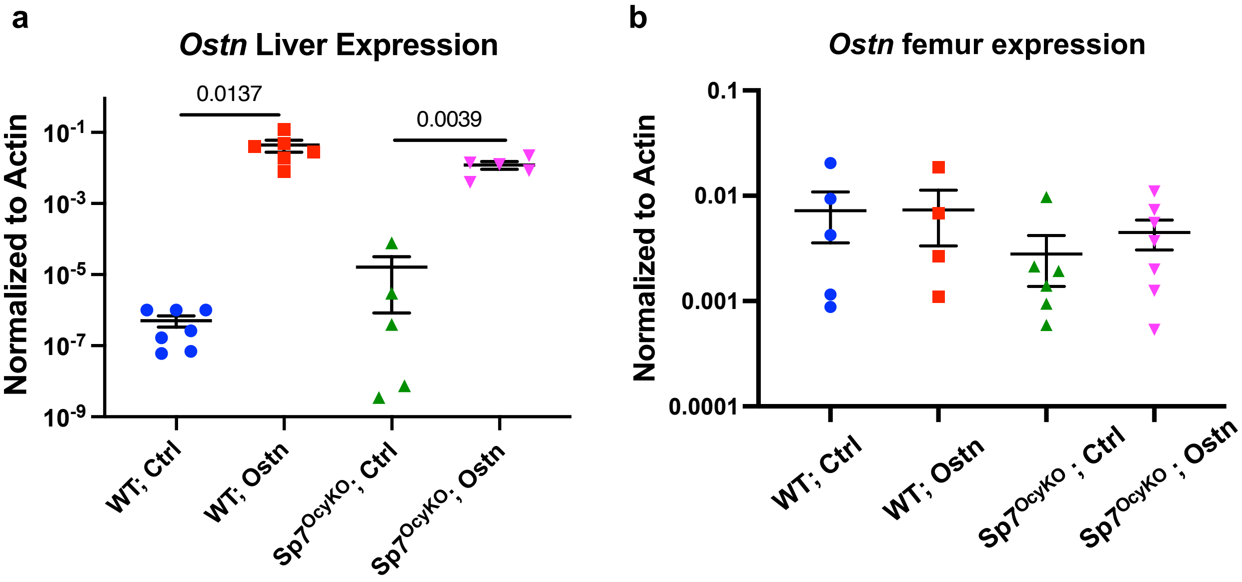

Fig. S9: RNA was isolated from liver (**a**) and femur (**b**) of mice infected with control AAV8 or AAV8-mOstn. Bone marrow was flushed out from femur before RNA isolation. Hepatic *Ostn* expression is very low in control mice, and robustly induced after AAV8-mOstn infection (note log10 y-axis scale for both graphs). Sp7 mutant mice show reduced cortical bone Ostn expression compared to control, but AAV8-mOstn treatment only increased hepatic Ostn expression levels. The color code for this figure is identical to those in Figures 5i-l, blue = WT + control AAV8, red = WT + AAV8-mOstn, green = Sp7 cKO + control AAV8, pink = Sp7 cKO + AAV8-mOstn. Each data point represents data obtained from a biologically independent animal. Data are presented as mean values +/- SEM. Two-sided student's t test was used to examine effects of AAV8-Ostn expression within each genotype. Exact p values are shown in the graph.

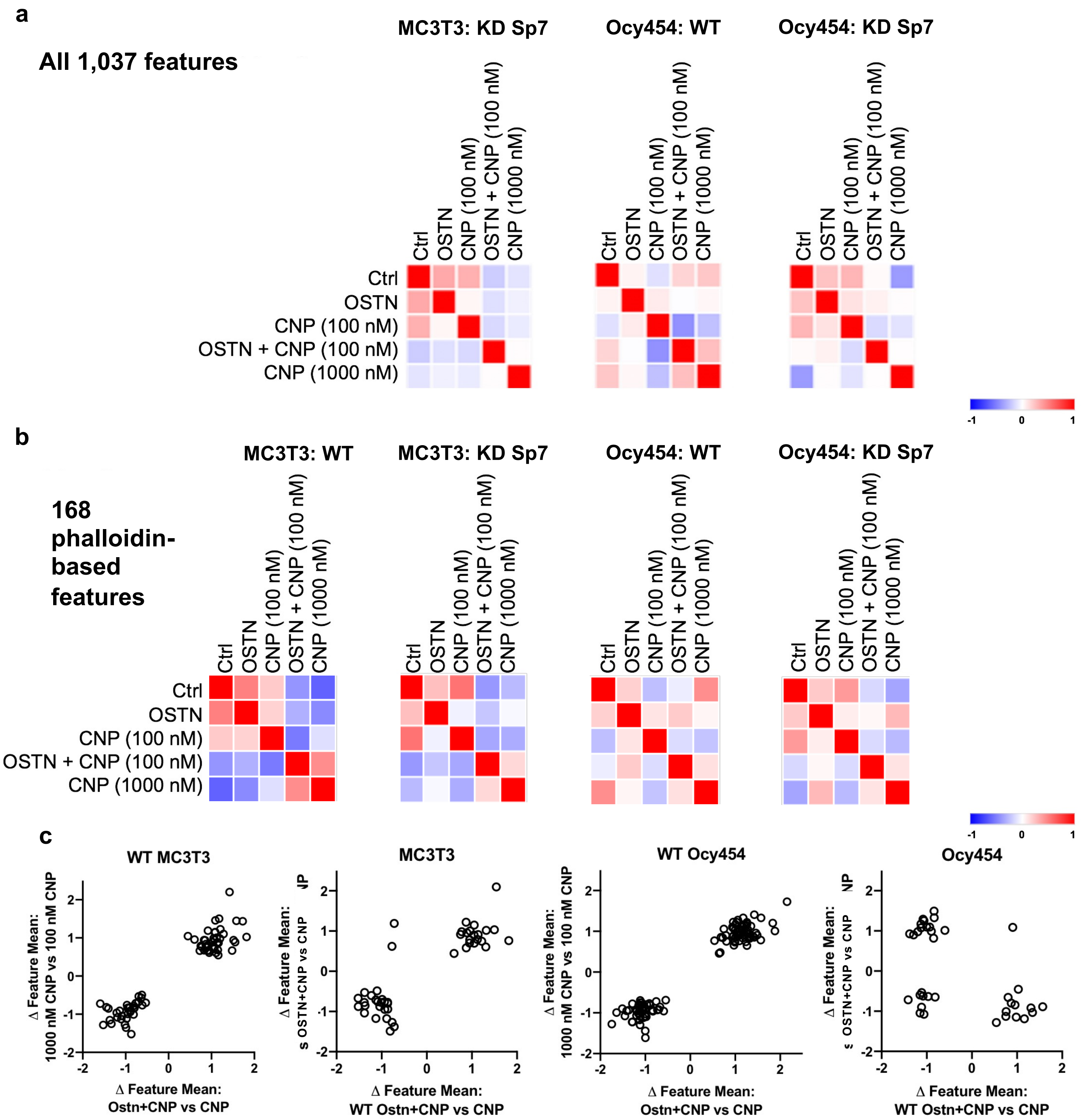

Fig. S10: (**a**) Similarity matrices for each cell type/genotype show Pearson correlations (6 median-collapsed replicates, 1037 features) after CNP and/or OSTN treatment. Red designates overall similarity and blue overall dissimilarity in Cell Painting feature measurements. (**b**) Similarity matrices for each cell type/genotype show Pearson correlations (6 median-collapsed replicates, 168 phalloidin-associated features) after CNP and/or OSTN treatment. Red designates overall similarity and blue overall dissimilarity in Cell Painting feature measurements. (**c**) Top markers differentially regulated by OSTN or high-dose CNP treatment were identified in each cell type/genotype. Top markers that were regulated by both treatments in WT cells and top markers that were regulated by OSTN in two cell genotypes are plotted here as the difference in mean normalized feature value between conditions.

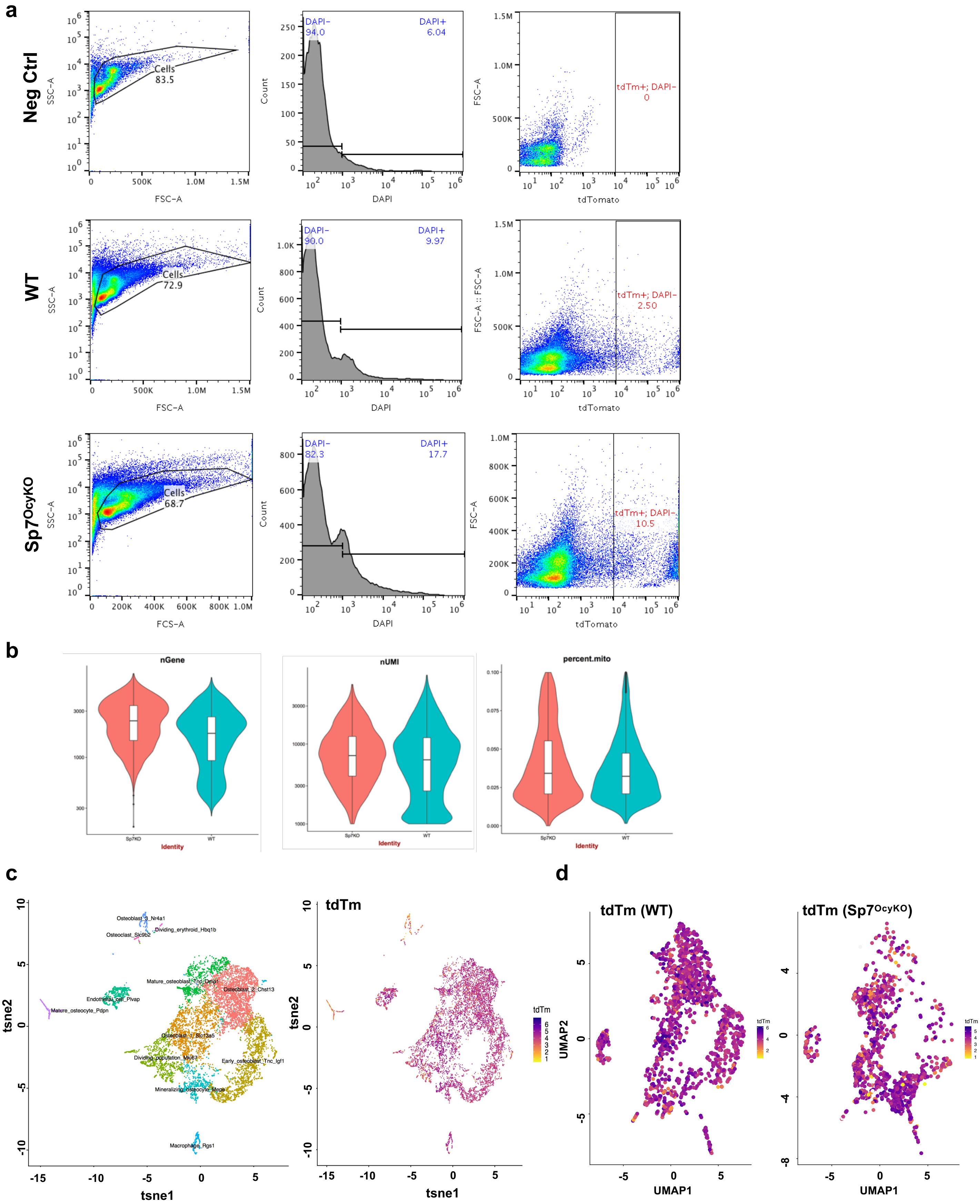
a
Neg Ctrl
WT
Sp7OcyKO
Cells 83.5
Cells 72.9
Cells 68.7
SSC-A
FSC-A
FCS-A
DAPI- 94.0
DAPI+ 6.04
DAPI- 90.0
DAPI+ 9.97
DAPI- 82.3
DAPI+ 17.7
Count
DAPI
tdTm+; DAPI- 0
tdTm+; DAPI- 2.50
tdTm+; DAPI- 10.5
FSC-A
FSC-A :: FSC-A
tdTomato
b
nGene
nUMI
percent.mito
Sp7KO
WT
Identity
c
Osteoblast_3_Nr4a1
Dividing_erythroid_Hbq1b
Osteoclast_Slc9b2
Mature_osteoblast_Tnc_Dmp1
Osteoblast_2_Chst13
Endothelial_cell_Plvap
Mature_osteocyte_Pdpn
Osteoblast_1_Slc13a5
Dividing_population_Mki67
Early_osteoblast_Tnc_Igf1
Mineralizing_osteocyte_Mepe
Macrophage_Rgs1
tsne1
tsne2
tdTm
d
tdTm (WT)
tdTm (Sp7OcyKO)
UMAP1
UMAP2
tdTm

Fig. S11: (**a**) Flow cytometry plots showing doublet exclusion and tdTomato expression in DAPI-negative cells obtained from fractions 5, 7, and 8 after serial collagenase/EDTA digestions and then sorted for single-cell RNA-sequencing. (**b**) Quality control metrics from WT and $Sp7^{OcyKO}$ libraries showing acceptable distribution of genes detected per cell, unique molecular identifiers per cell, and mitochondrial read abundance. Violin plots show data distribution across all cells from the indicated libraries. The 25% percentile/median/75% percentile values for each parameter are: nGene (Sp7 cKO) 1494/2379/3419, nGene (WT) 927/1771/2611, nUMI (Sp7 cKO) 3973/7175/12441, nUMI (WT) 2586/6362/12024, pcMITO (Sp7 cKO) 0.034/0.055/0.099, pcMITO (WT) 0.032/0.047/0.087. (**c**) Left: clustering of all cells sequenced reveals main populations of osteoblast-lineage cells, along with other cell types including endothelial cells, macrophages, osteoclasts, and dividing erythroid cells. Right: tdTomato expression was mapped to all cells sequenced. Of note, some non-osteoblast lineage cells (endothelial cells, macrophages) do express detectable tdTomato mRNA. These clusters plus osteoclasts and dividing erythroid cells were excluded from future analyses. (**d**) tdTomato expression in remaining osteoblast-lineage cells shows comparable expression levels of this Dmp1-Cre-dependent reporter in WT and $Sp7^{OcyKO}$ cells.

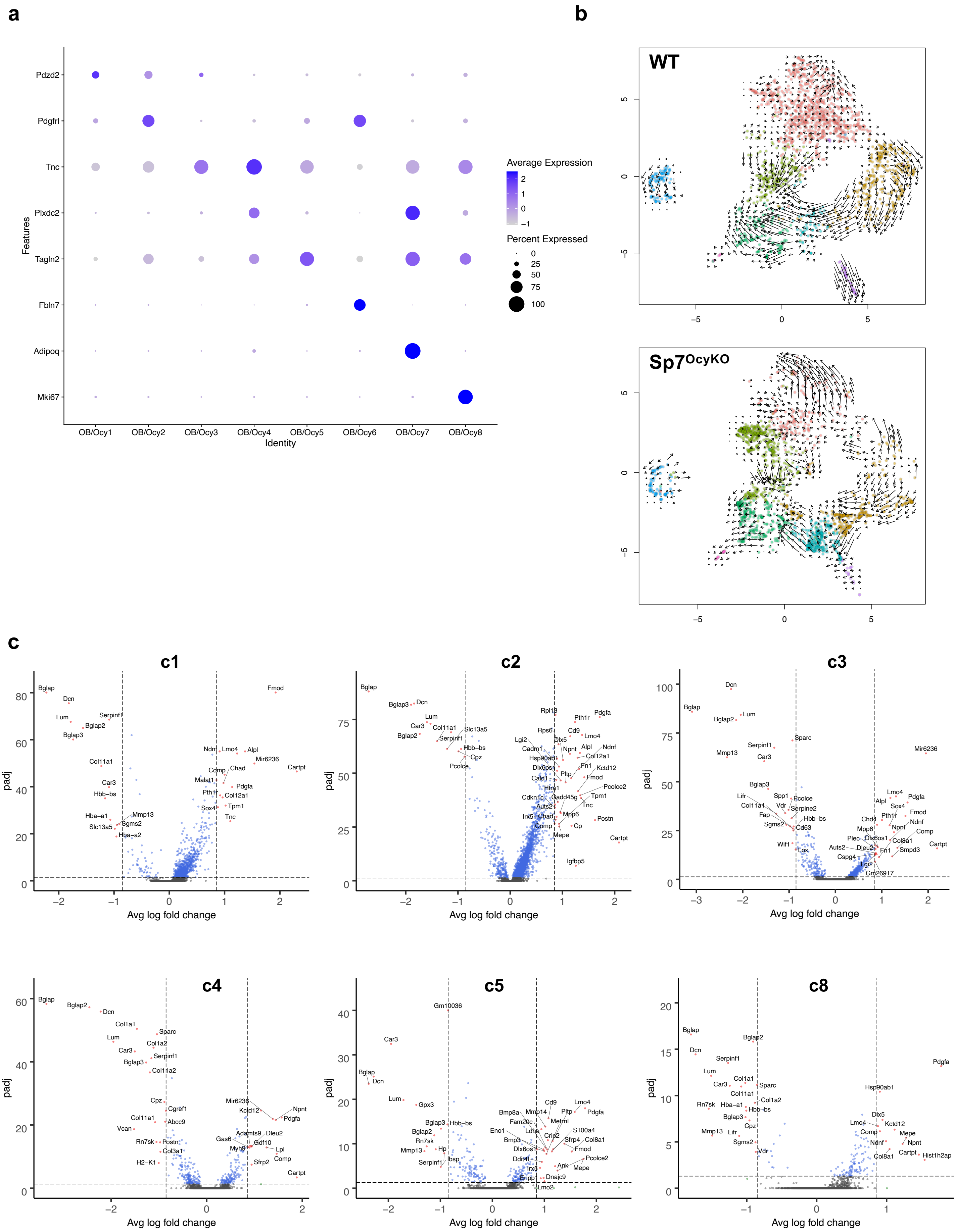
a
Pdzd2
Pdgfrl
Tnc
Plxdc2
Tagln2
Fbln7
Adipoq
Mki67
Features
OB/Ocy1
OB/Ocy2
OB/Ocy3
OB/Ocy4
OB/Ocy5
OB/Ocy6
OB/Ocy7
OB/Ocy8
Identity
Average Expression
Percent Expressed
b
WT
Sp7^OcyKO
c
c1
c2
c3
c4
c5
c8
padj
Avg log fold change
Bglap
Dcn
Lum
Serpinf1
Bglap2
Bglap3
Col11a1
Car3
Hbb-bs
Hba-a1
Mmp13
Sgms2
Slc13a5
Hba-a2
Fmod
Ndnf
Lmo4
Alpl
Mir6236
Cartpt
Comp
Chad
Malat1
Pdgfa
Pth1r
Col12a1
Sox4
Tpm1
Tnc
Rpl13
Pth1r
Cd9
Postn
Cp
Mepe
Igfbp5
Sparc
Gm26917
Gm10036
Hsp90ab1
Hist1h2ap

Fig. S12: (**a**) Dotplot showing the expression of top1 marker in each cluster. (**b**) Velocyto trajectory analysis in control and *Sp7*^{OcyKO} libraries shows lack of orderly progression to the terminal osteocyte stage (cluster 6 in purple) in *Sp7* mutant mice. Red arrows again indicate apparent differentiation arrest in c3 and c5. See also Fig. 6n. (**c**) Cluster-specific differential gene expression volcano plots for indicated clusters. The x-axis shows log2 fold change (*Sp7* mutant versus WT) and the y-axis shows the adjusted p value. A Wilcoxon rank-sum test with an equal number of cells per library was used for statistical analysis.

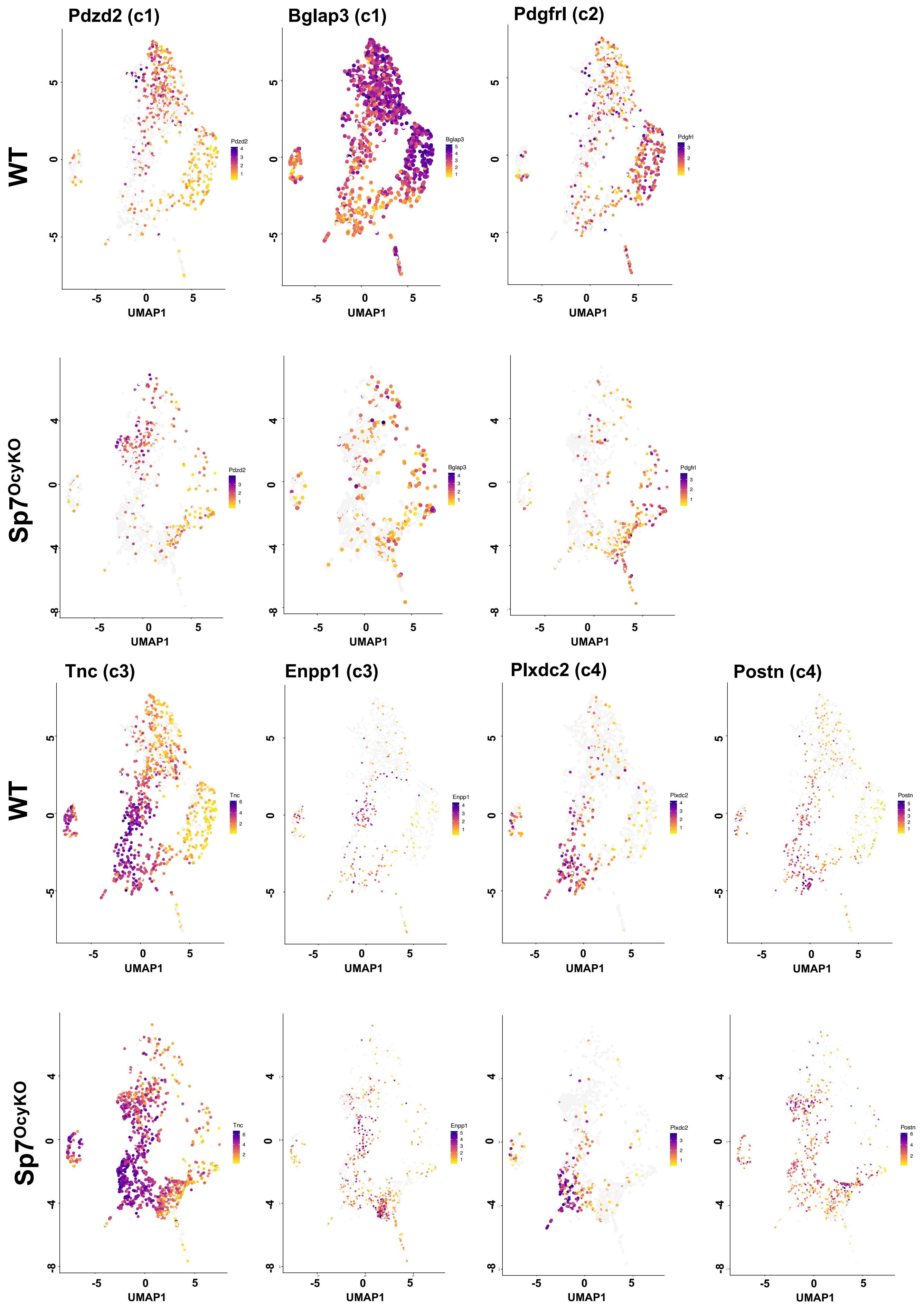

Pdzd2 (c1)
Bglap3 (c1)
Pdgfrl (c2)
WT
Sp7OcyKO
Tnc (c3)
Enpp1 (c3)
Plxdc2 (c4)
Postn (c4)
WT
Sp7OcyKO
UMAP1

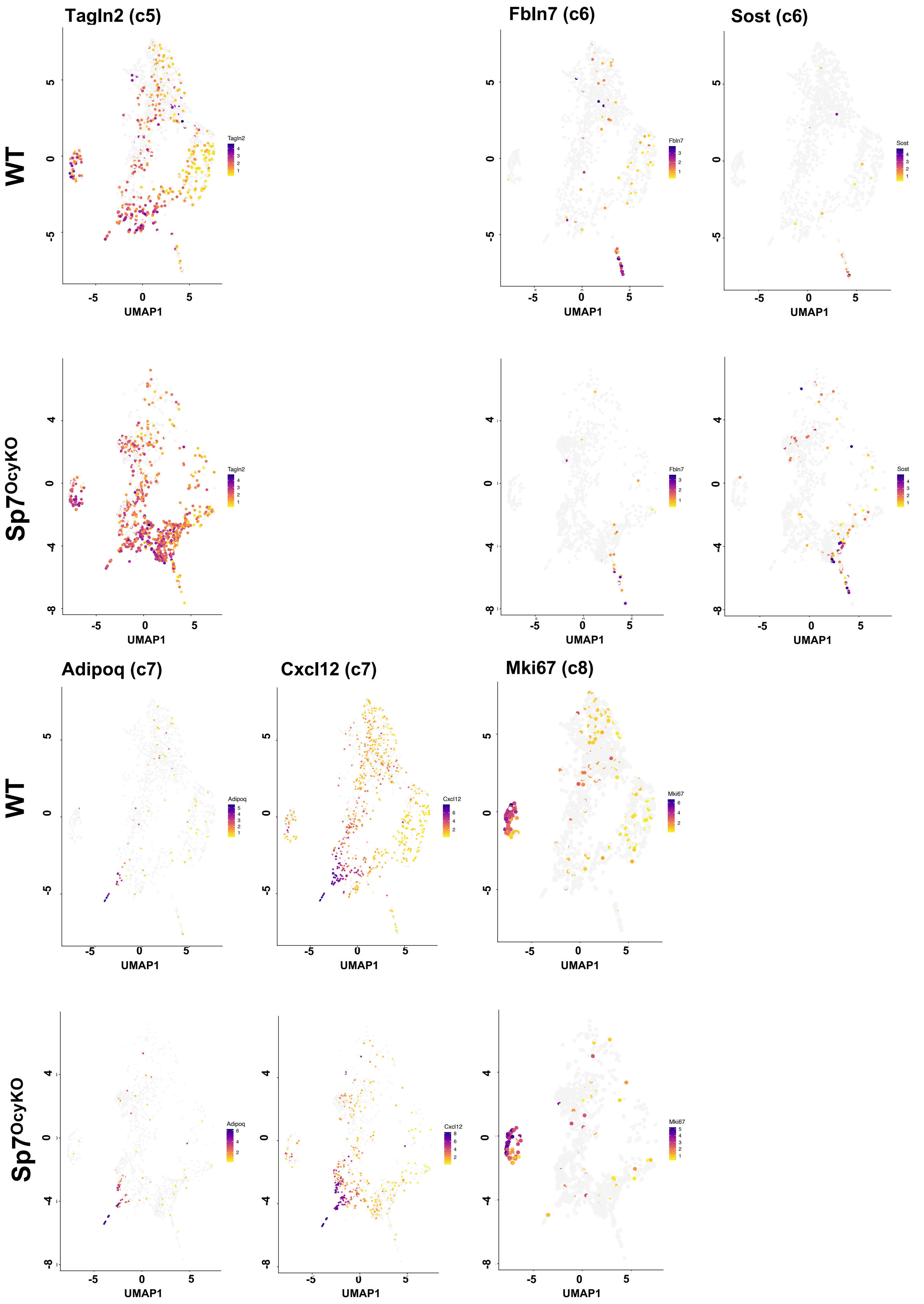

Tagln2 (c5)
Fbln7 (c6)
Sost (c6)
WT
Sp7OcyKO
Adipoq (c7)
Cxcl12 (c7)
Mki67 (c8)
WT
Sp7OcyKO
UMAP1
Tagln2
Fbln7
Sost
Adipoq
Cxcl12
Mki67

Fig. S13: Feature plots showing the expression of 1) top1 marker and 2) one known osteo-lineage marker of each cluster (c1-c8).

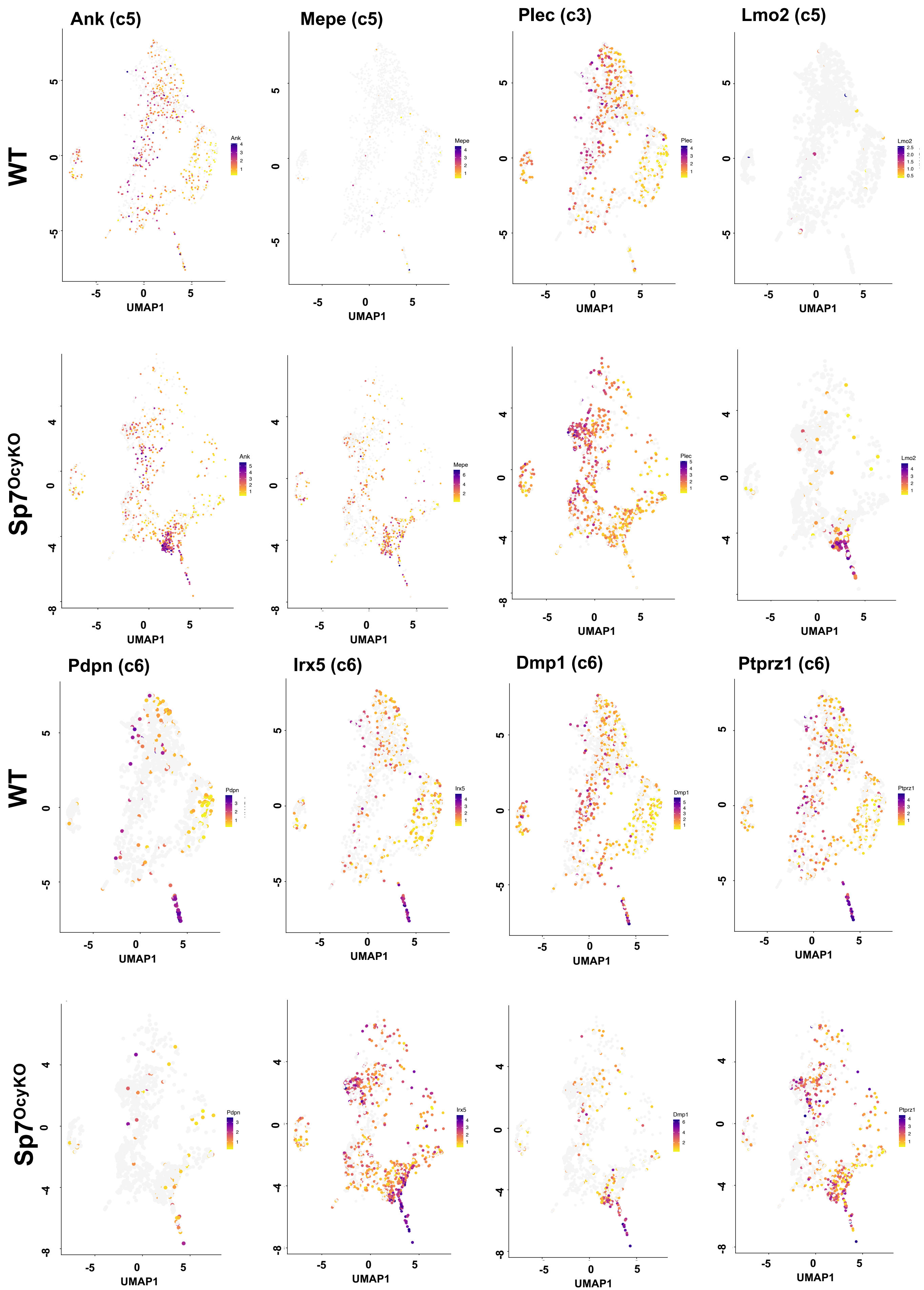
Ank (c5)
Mepe (c5)
Plec (c3)
Lmo2 (c5)
WT
Sp7OcyKO
Pdpn (c6)
Irx5 (c6)
Dmp1 (c6)
Ptprz1 (c6)
WT
Sp7OcyKO
UMAP1

Fig. S14: Feature plots showing relative transcript abundance of indicated genes. Top: expression of mineralization-related genes and (Ank, Mepe) and the expression of genes that mark c3 and c5 (*Plec, Lmo2*). Bottom: the expression of canonical osteocyte (*Dmp1, Pdpn*) marker genes and the expression of genes that have significant changes in *Sp7* mutant libraries (*Irx5, Ptprz1*). In WT libraries, osteocyte marker expression is restricted to cluster 6, while dysregulated expression of *Sost*, *Irx5*, and *Ptprz1* is noted in *Sp7* mutant libraries, especially in *Lmo2*-expressing cells in cluster 5 and *Plec*-expressing cells in cluster 3. In contrast, other terminal osteocyte markers (*Pdpn* and *Fbln7*) are restricted to osteocytes in control mice and essentially absent in *Sp7* mutants.

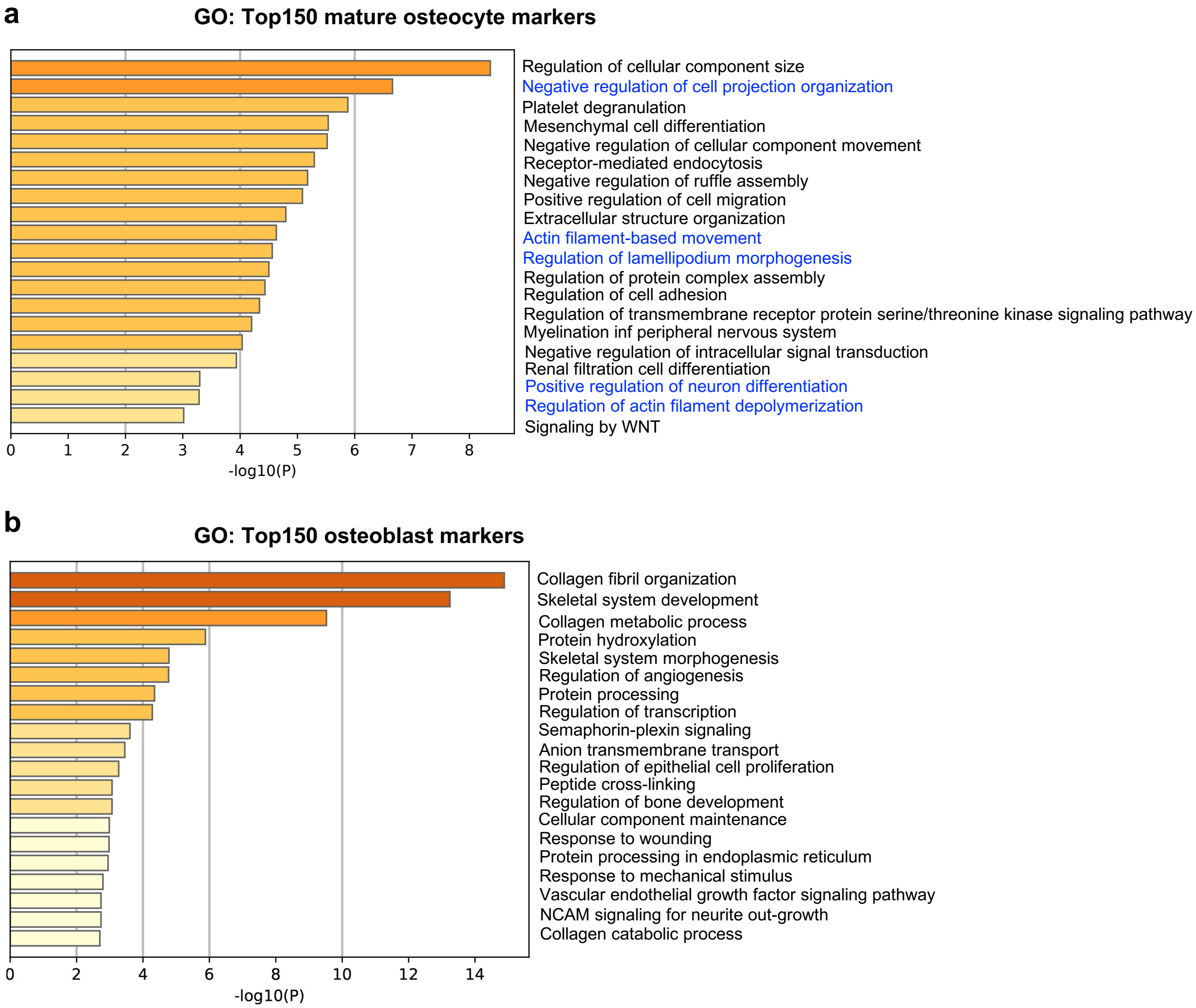

Fig. S15: (**a-b**) GO enrichment terms of top150 mature osteocyte markers derived from cluster 6 and top150 canonical osteoblast markers derived from cluster 1+2. Fisher's exact test with correction for multiple hypothesis testing was used.

a

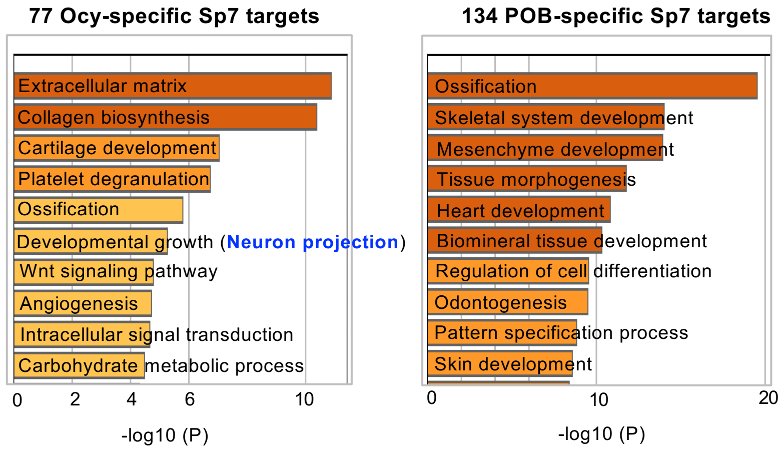

b

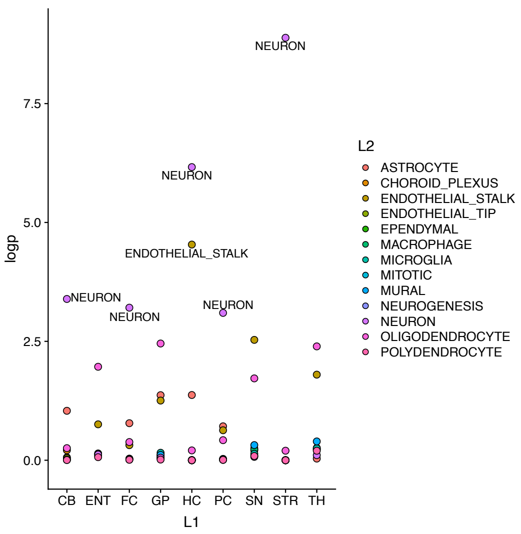

Fig. S16: (**a**) GO enrichment of 77 osteocyte-specific (left) and 134 primary osteoblast-specific (right) Sp7 targets. Fisher's exact test with correction for multiple hypothesis testing was used. (**b**) Enrichment score of 77 gene set expression in cell types across nine mouse brain regions. CB - cerebellum, ENT - entorhinal cortex, FC - frontal cortex, GP - globus pallidus, HC - hippocampus, PC -parietal cortex, SN - substantia nigra, STR - striatum, TH - thalamus. Across multiple brain regions, neurons show relative enrichment of this gene set versus other non-neuronal cell types. Wilcoxon rank sum test was used to calculate enrichment scores and p values.

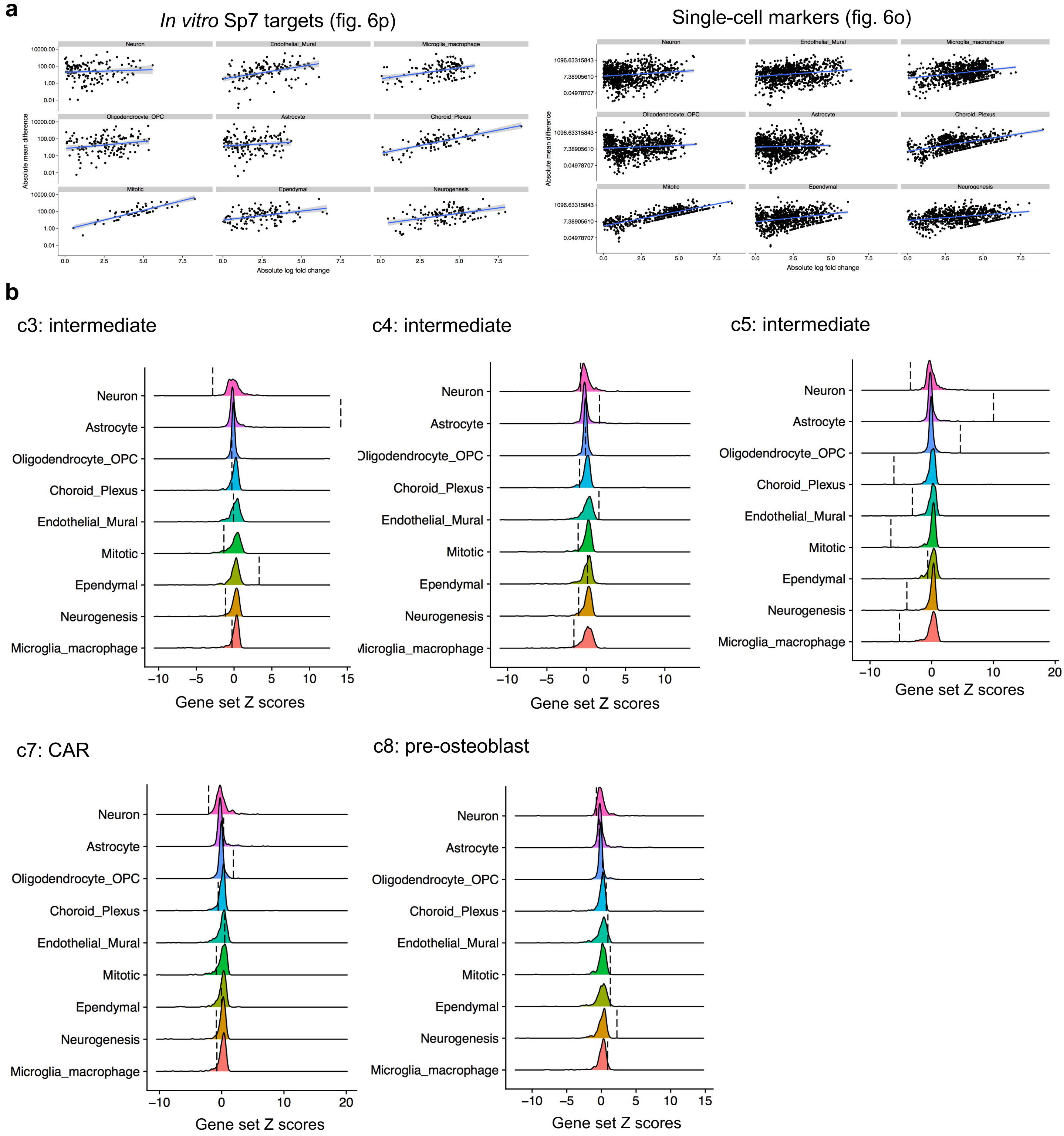

Fig. S17: (**a**) Correlation between brain cell type mean difference and log_2FC of *in vitro Sp7* targets (left, Fig. 6p) and top150 single-cell RNA-seq markers (right, Fig. 6o). See Methods for details. (**b**) The expression of top150 markers from other osteo-lineage clusters (c3-c5, c7-8) was analyzed in a mouse brain single-cell RNA-seq atlas. None of them show significant enrichment in neurons compared to other brain cell types. OPC: Oligodendrocyte progenitor cell; Mitotic: Mitotic cells, Neurogenesis: Neurogenesis-associated cells.

a

Group 1

Gene	Ctrl	$Sp7^{OcyKO}$
Tchh	C1+2+6	↓1+2+6; ↑3+5
Ngef	C1+2+6	↓1+2+6; ↑3+5
Coro6	C1+2+6	↓1+2+6; ↑5
Cryab	C1+2+6	↓1+2+6; ↑3+5
Kank1	C1+2+6	↓1+2+6; ↑3+5
Rapgef4	C1+2+6	↓1+2+6; ↑3+5
Mgll	All	↓1+2+6; ↑3+5
Frzb	C1+2+4	↓1+2; 5↑

Group 2

Gene	Ctrl	$Sp7^{OcyKO}$
Sost	C6	↓6; 3+5↑
Ackr3	C6	↓6; 3+5↑
Ptprr	C6	↓6

b

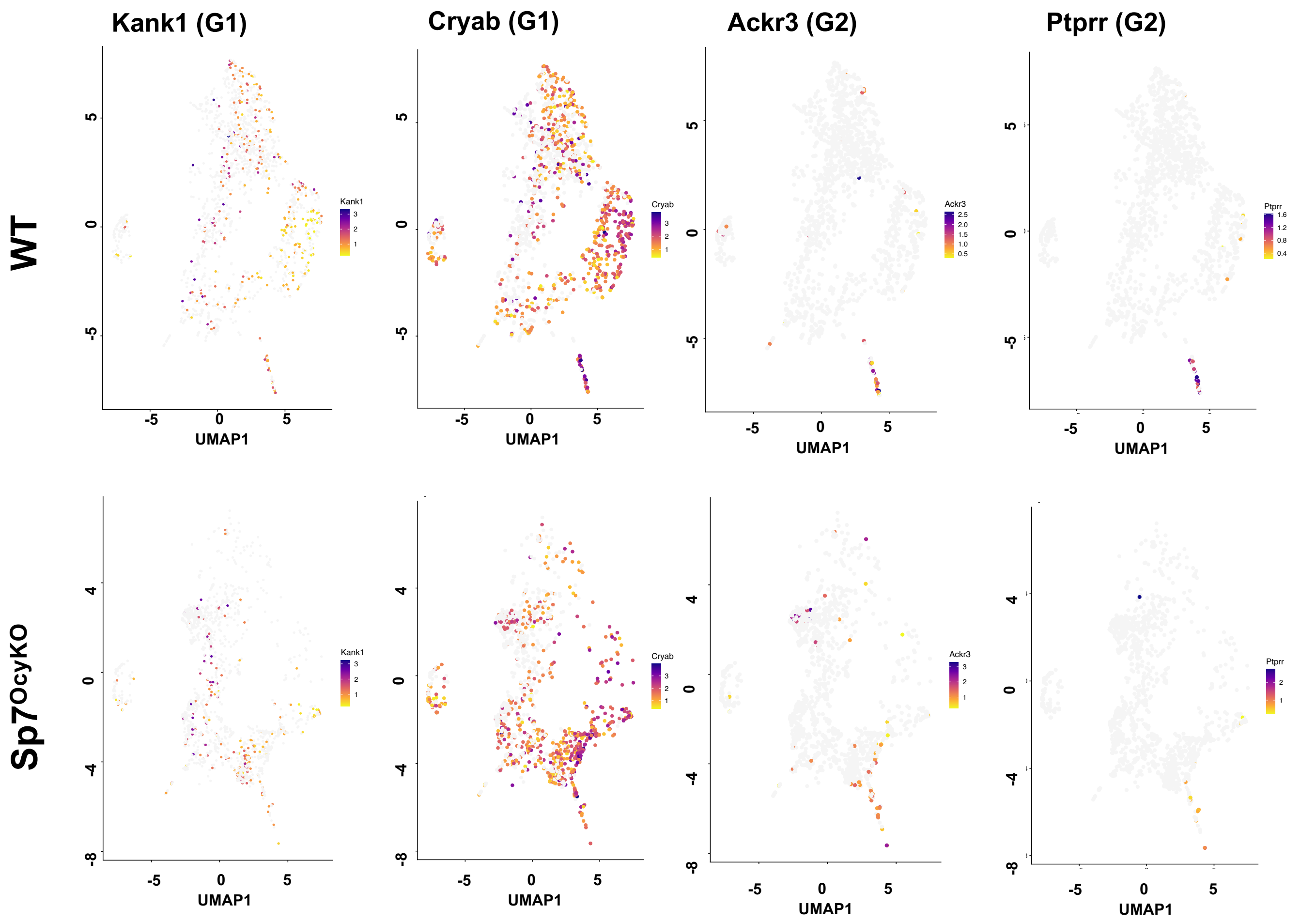

Fig. S18: (**a**) Ocy454 cell RNA-seq and ChIP-seq datasets identified a group of neuronal-like genes whose expression is directly regulated by Sp7 in osteocytes. Here, the expression patterns of those genes in Dmp1-Cre-labelled cells from bone is examined. One group of genes are expressed in canonical osteoblast and mature osteocyte clusters (c1+2+6). One group of genes show restricted expression in mature osteocyte cluster (c6). (**b**) feature plots of the genes shown in (a). See text for further details.

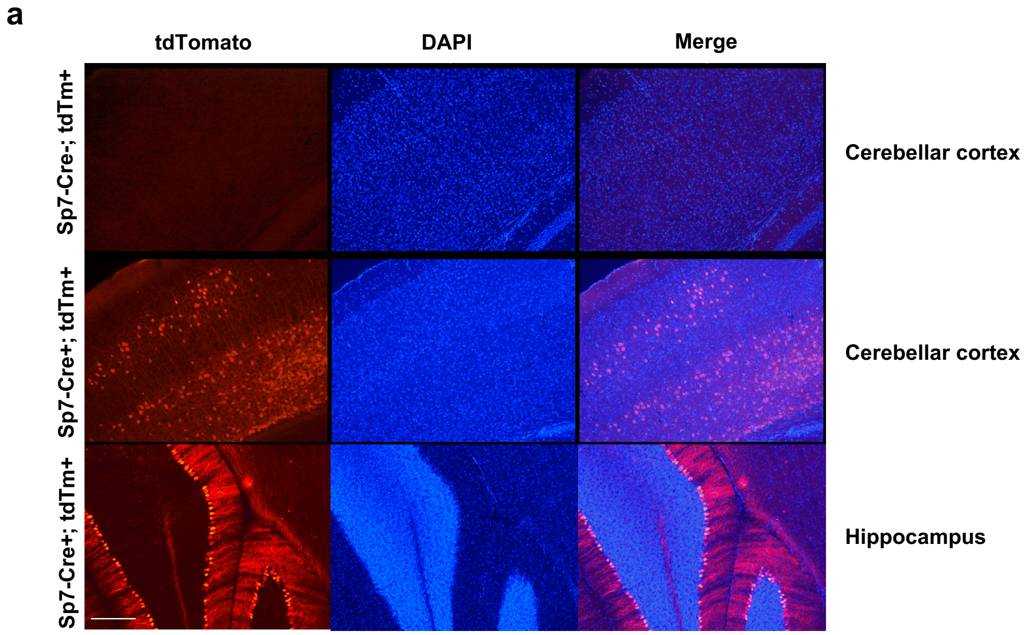

Fig. S19: (**a**) 8 week old control and Sp7-Cre ; tdTomato^{LSL} mice were analyzed for expression of tdTomato in cerebellar cortex (top) and hippocampus. No tdTomato-cells were present in Sp7-Cre-negative mice. In contrast, in Sp7-Cre-positive animals, we note cells with neuronal morphology in these brain regions. Representative images from three mice per genotype are shown. Scale bar = 100 µm.

a

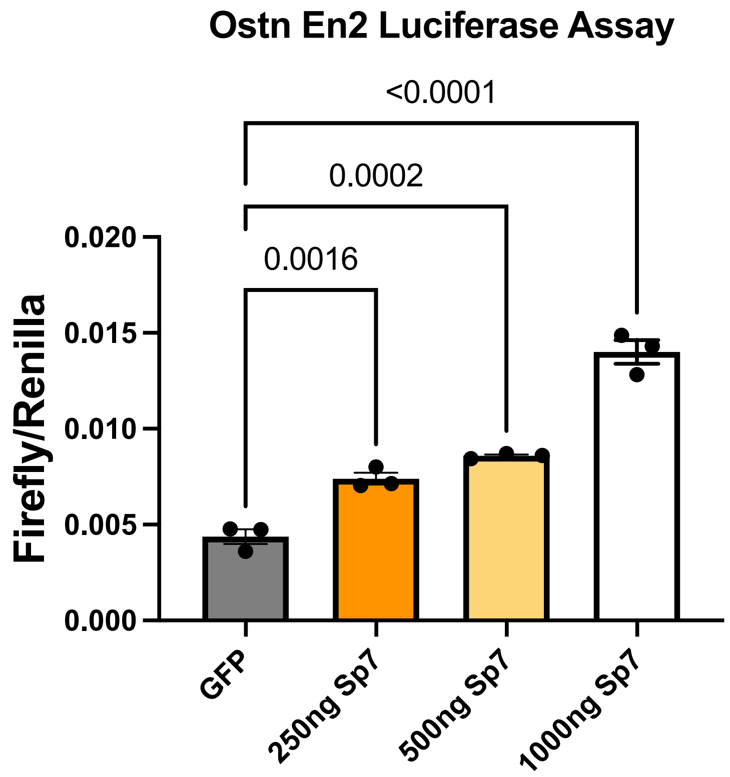

Fig. S20: (**a**) Luciferase assay in 293T cells showing effects of murine *Sp7* overexpression on *osteocrin* enhancer 2 activity. 1-way ANOVA was performed followed by Dunnett's multiple comparisons test to compare each dose of murine Sp7 cDNA versus GFP control. p values for the indicated comparison are shown on the graph. n=3 biologically independent samples were analyzed. Data are presented as mean values +/- SEM.
