# Supplementary material for: Control of osteocyte dendrite formation by Sp7 and its target gene osteocrin
Source: Nat Commun. 2021 Nov 1;12:6271. doi: 10.1038/s41467-021-26571-7 (PMC8560803; doi:10.1038/s41467-021-26571-7)
Supplement: Supplementary file 3 — Description of Additional Supplementary Files [file 41467_2021_26571_MOESM3_ESM.docx]

Description of Additional Supplementary Files

**Title: Supplementary Data 1.**

**Description:** MicroCT and histomorphometry results

**Title: Supplementary Data 2.**

**Description:** Sp7 over-expression, knockdown and 3D culture RNA-seq results

**Title: Supplementary Data 3.**

**Description:** Sp7 ChIP-seq results

**Title: Supplementary Data 4.**

**Description:** ChIP-seq enrichment motif results

**Title: Supplementary Data 5.**

**Description:** Cell type-specific genes associated with Sp7

**Title: Supplementary Data 6.**

**Description:** 77 osteocyte-specific Sp7 targets

**Title: Supplementary Data 7.**

**Description:** Ostn rescue RNA-seq results

**Title: Supplementary Data 8.**

**Description:** Cell Painting data files

**Title: Supplementary Data 9.**

**Description:** Single cell RNA-seq output files

**Title: Supplementary Data 10.**

**Description:** Oligonucleotide sequences used
