# Supplementary material for: Control of osteocyte dendrite formation by Sp7 and its target gene osteocrin
Source: Nat Commun. 2021 Nov 1;12:6271. doi: 10.1038/s41467-021-26571-7 (PMC8560803; doi:10.1038/s41467-021-26571-7)
Supplement: Supplementary file 14 — Reporting Summary [file 41467_2021_26571_MOESM14_ESM.pdf]

nature research

Corresponding author(s): Marc Wein

Last updated by author(s): Oct 8, 2021

Reporting Summary

Nature Research wishes to improve the reproducibility of the work that we publish. This form provides structure for consistency and transparency in reporting. For further information on Nature Research policies, see our Editorial Policies and the Editorial Policy Checklist.

Statistics

For all statistical analyses, confirm that the following items are present in the figure legend, table legend, main text, or Methods section.

n/a	Confirmed	
☐	☒	The exact sample size (*n*) for each experimental group/condition, given as a discrete number and unit of measurement
☐	☒	A statement on whether measurements were taken from distinct samples or whether the same sample was measured repeatedly
☐	☒	The statistical test(s) used AND whether they are one- or two-sided<br>*Only common tests should be described solely by name; describe more complex techniques in the Methods section.*
☐	☒	A description of all covariates tested
☐	☒	A description of any assumptions or corrections, such as tests of normality and adjustment for multiple comparisons
☐	☒	A full description of the statistical parameters including central tendency (e.g. means) or other basic estimates (e.g. regression coefficient) AND variation (e.g. standard deviation) or associated estimates of uncertainty (e.g. confidence intervals)
☐	☒	For null hypothesis testing, the test statistic (e.g. *F*, *t*, *r*) with confidence intervals, effect sizes, degrees of freedom and *P* value noted<br>*Give P values as exact values whenever suitable.*
☒	☐	For Bayesian analysis, information on the choice of priors and Markov chain Monte Carlo settings
☒	☐	For hierarchical and complex designs, identification of the appropriate level for tests and full reporting of outcomes
☐	☒	Estimates of effect sizes (e.g. Cohen's *d*, Pearson's *r*), indicating how they were calculated

Our web collection on statistics for biologists contains articles on many of the points above.

Software and code

Policy information about availability of computer code

Data collection	BD Sorp 8 Laser LSR II (BDBiosciences); Cell Ranger version 3.1.0 (10X Genomics); SH800s Cell Sorter (Sony); µCT 40 (Scanco Medical AG, Brüttisellen, Switzerland)
Data analysis	Image J Imaging Software version 1.50i (NIH); GraphPad Prism version 8; Imaris version 7.4.2 (Bitplane); FlowJo Software version 10 (TreeStar); STAR version 2.7.2b; HTSeq version 0.9.1; EdgeR version 3.24.0 (Bioconductor); ggplot2/tidyverse; clusterProfiler; Rank-Rank Hypergeometric Overlap 2; OsteoMeasure (OsteoMetrics); EnhancedVolcano (Bioconductor); FastQC (version 0.11.8); Bowtie2 (version 2.4.0); MACS2 (version 2.2.7.1); BEDTools (version 22.8); ChIPseeker (version 3.13); GREAT (version 4); DREME (version 5.4.1); deepTools version 2.5.0; Cell Ranger Analysis version 3.1.0; LIGER (version 1.0); Monocle3 (release 3.13); velocyto (version 0.6); MAGMA (version 1.08); DropViz (original release); Morpheus (version 5.3.2); Metascape (version 3.5)

For manuscripts utilizing custom algorithms or software that are central to the research but not yet described in published literature, software must be made available to editors and reviewers. We strongly encourage code deposition in a community repository (e.g. GitHub). See the Nature Research guidelines for submitting code & software for further information.

Data

Policy information about availability of data

All manuscripts must include a data availability statement. This statement should provide the following information, where applicable:

- Accession codes, unique identifiers, or web links for publicly available datasets
- A list of figures that have associated raw data
- A description of any restrictions on data availability

The new RNA-seq, ChIP-seq and scRNA-seq data has been deposited in NCBI's Gene Expression Omnibus (GEO) (GSE154719). The authors declare that all other data supporting the findings of this study are available within the article and its supplementary information files. Source data are provided with this paper.

Field-specific reporting

Please select the one below that is the best fit for your research. If you are not sure, read the appropriate sections before making your selection.

☒ Life sciences ☐ Behavioural & social sciences ☐ Ecological, evolutionary & environmental sciences

For a reference copy of the document with all sections, see nature.com/documents/nr-reporting-summary-flat.pdf

Life sciences study design

All studies must disclose on these points even when the disclosure is negative.

Sample size	No statistical method was used to predetermine sample size. We chose the numbers of mice to study based on our prior experience (PMID 34160349 for example) so as to provide sufficient power for statistical comparison.
Data exclusions	Exclusions were based on loss or damage of samples during tissue processing. This only occurred for only one in the WT/ctrl group for the experiment shown in Figure 5.
Replication	For all data presented in the manuscript, we examined at least three independent biological samples (three different mice) to ensure the reproducibility. For each series of the experiments, all attempts at replication were successful.
Randomization	The experiments were not randomized. Mice were allocated to particular groups (wild-type, Sp7 KO) based on results of PCR-genotyping performed around two weeks after birth on an alternating basis. Covariates were controlled by considering multiple factors, such as genotypes and general phenotypical data (i.e. body weight). On principle, we did not observe any particular difference among groups.
Blinding	Data was blinded with respect to group allocation during histological, phalloidin staining, silver staining and TUNEL analysis.

Reporting for specific materials, systems and methods

We require information from authors about some types of materials, experimental systems and methods used in many studies. Here, indicate whether each material, system or method listed is relevant to your study. If you are not sure if a list item applies to your research, read the appropriate section before selecting a response.

Materials & experimental systems

n/a	Involved in the study	
☐	☒	Antibodies
☐	☒	Eukaryotic cell lines
☒	☐	Palaeontology and archaeology
☐	☒	Animals and other organisms
☐	☒	Human research participants
☒	☐	Clinical data
☒	☐	Dual use research of concern

Methods

n/a	Involved in the study	
☐	☒	ChIP-seq
☐	☒	Flow cytometry
☒	☐	MRI-based neuroimaging

Antibodies

Antibodies used

Abcam
Rabbit anti-Sp7 (Cat# ab22552; RRID:AB_2194492), dilution 1:1000

Cell Signaling Technology
Rabbit anti-DYKDDDDK Tag (Cat# 2368; RRID:AB_2217020), dilution 1:1000
Rabbit anti-GAPDH (Cat# 2118; RRID:AB_561053), dilution 1:1000
Rabbit anti-β-tubulin (Cat# 2146; RRID:AB_2210545), dilution 1:2000
Rabbit anti-phospho-Erk1/2 (Cat #9101, RRID:AB_330744), dilution 1:2000
Rabbit anti-Erk1/2 (Cat #4695, RRID:AB_390779), dilution 1:1000

InvitrogenTM
Dynabeads™ M-280 Sheep anti-mouse IgG (Cat# 11201D)

Sigma Aldrich
Mouse anti-FLAG M2 (Cat# F1804), dilution 1:1000

Validation

More detailed information about these antibodies is available on these manufacturers' websites. Specifically, all antibodies used have been validated for mouse antigens.

Eukaryotic cell lines

Policy information about cell lines

Cell line source(s)	HEK293T from ATCC, MC3T3-E1 from ATCC, Ocy454 cells from PMID 25953900
Authentication	Ocy454 cells were authenticated based upon robust sclerostin up-regulation following growth for >7 days at 37C. 293T and MC3T3-E1 cells were not authenticated.
Mycoplasma contamination	All lines tested negative for mycoplasma contamination
Commonly misidentified lines (See ICLAC register)	No commonly misidentified lines were used.

Animals and other organisms

Policy information about studies involving animals; ARRIVE guidelines recommended for reporting animal research

Laboratory animals	Dmp1-Cre transgenic mice (RRID: MGI:3784520) and Ai14 Cre-dependent tdTomato reporter (JAX007914) were intercrossed. Floxed Sp7 mice were kindly provided by Dr. Benoit de Crombrugghe. Dmp1-Cre; tdTm+ mice were crossed with Sp7 fl/+ mice to generate Sp7OcyKO (Dmp1-Cre; Sp7fl/fl; tdTm+) and control (Dmp1-Cre; Sp7+/+; tdTm+) mice. Genotypes were determined by PCR using primers listed in table S8. All mouse strains were backcrossed to C57BL/6J for at least 4 generations. Both males and females were included in this study. Mice were analyzed at 6-8 weeks of age.
Wild animals	no wild animals were used.
Field-collected samples	no field collected samples were used in the study.
Ethics oversight	All procedures involving animals were performed in accordance with guidelines issued by the Institutional Animal Care and Use Committees (IACUC) in the Center for Comparative Medicine at the Massachusetts General Hospital and Harvard Medical School under approved Animal Use Protocols (2019N000201).

Note that full information on the approval of the study protocol must also be provided in the manuscript.

Human research participants

Policy information about studies involving human research participants

Population characteristics	No new human studies were performed as part of this manuscript. De-identified biopsy samples collected from previous studies (PMID 29382611 and PMID 17968492) were analyzed ex vivo.
Recruitment	No new human subjects were recruited as part of this manuscript.
Ethics oversight	The Sydney Children's Hospital Network Human Research Ethics Committee (CCR2017/19) approved all studies regarding subjects with Sp7 mutations, as described in PMID 29382611. The study protocol for collection of control subject samples was approved by the ethics committee of the University of Sao Paulo (664/97), as detailed in detail in PMID 17968492.

Note that full information on the approval of the study protocol must also be provided in the manuscript.

ChIP-seq

Data deposition

- [x] Confirm that both raw and final processed data have been deposited in a public database such as GEO.
- [x] Confirm that you have deposited or provided access to graph files (e.g. BED files) for the called peaks.

Data access links *May remain private before publication.*	https://www.ncbi.nlm.nih.gov/geo/query/acc.cgi?acc=GSE154719
Files in database submission	Ocy454_Input_R1_001.fastq.gz<br>Ocy454_Input_R2_001.fastq.gz<br>Ocy454_Sp7_ChIPseq_R1_001.fastq.gz<br>Ocy454_Sp7_ChIPseq_R2_001.fastq.gz<br>Ocy454_Sp7_ChIPseq_narrow_peak.bed.gz<br>Ocy454_Sp7_ChIPseq_narrow_peak.bigwig
Genome browser session (e.g. UCSC)	https://genome.ucsc.edu/cgi-bin/hgTracks?db=mm9&lastVirtModeType=default&lastVirtModeExtraState=&virtModeType=default&virtMode=0&nonVirtPosition=&position=chr12%3A57795963%2D57815592&hgsid=929664579_sUg0sQFFkQBzLjgh5hr06jJyOEkW

Methodology

Replicates	All ChIP-seq experiments were done in biological duplicates.
Sequencing depth	Each library was sequenced on HiSeq X (Illumina) platforms. Sequencing were done to achieve more than 20 million reads per biological replicates.
Antibodies	Dynabeads™ M-280 Sheep anti-mouse IgG (InvitrogenTM, Cat# 11201D)
Peak calling parameters	ChIP and input control libraries were aligned to the mouse genome (mm9) using Bowtie2 with parameters specified to report the best alignment allowing no more than three mismatches within either the first twenty bases on the high-quality end of the read and excluding reads that aligned to more than one location in the genome (--best -l 20 -n 3 -m 1). Peaks were identified using MACS2 with default parameters except for the effective genome size set for mouse (-g mm).
Data quality	Sequencing read quality was evaluated using FastQC (http://www.bioinformatics.bbsrc.ac.uk/projects/fastqc/). A low quality read filter was then applied in which no reads with more than six bases with a minimum Phred quality score of twenty were retained.
Software	FastQC; Bowtie2; MACS2; BEDTools; ChIPseeker; GREAT; DREME; deepTools version 2.5.0

Flow Cytometry

Plots

Confirm that:

- [x] The axis labels state the marker and fluorochrome used (e.g. CD4-FITC).
- [x] The axis scales are clearly visible. Include numbers along axes only for bottom left plot of group (a 'group' is an analysis of identical markers).
- [x] All plots are contour plots with outliers or pseudocolor plots.
- [x] A numerical value for number of cells or percentage (with statistics) is provided.

Methodology

Sample preparation	For cells that were stained with phalloidin (Abcam, ab176753), cell pellets were resuspended with 2% FACS buffer (phosphate buffered saline plus 2% heat inactivated fetal bovine serum) and then filtered through round bottom tubes with cell strainer cap (Falcon®, 70 µm). For cells stained with Annexin V and EdU, cells were filtered through round bottom tubes with cell strainer cap (Falcon®, 70 um).<br>For scRNA-seq sample preparation, mice were sacrificed and tibiae, femura, and calvariae were dissected. Soft tissue was removed through scraping, and the epiphysis was cut off. Bone marrow cells were flushed out with cold PBS using a syringe. Bones were cut into 1- to 2-mm lengths and subjected to 8 serial digestions. Each digestion took place in 5 ml solution in a 15 ml centrifuge tube on the thermomixer set at 35ºC/500 rpm. Bone fragments were washed in PBS between digestions. The final three collagenase fractions were collected by centrifuging the supernatant at 4ºC/300 rcf/8 min. Cell pellets were resuspended with 2% FACS buffer containing RNase inhibitor and were filtered through a 100 µm cell strainer.
Instrument	BD Sorp 8 Laser LSR II (BDBiosciences); SH800s Cell Sorter (Sony)
Software	FlowJo Software version 10 (TreeStar)
Cell population abundance	Gating strategy shown in Supplemental Figure 11a demonstrates abundance of distinct cell poopulations.
Gating strategy	Dead cells, debris, doublets and triplets were excluded by FSC, SSC and DAPI. Negative 'unstained' control samples were always used as a reference to determine the demarcation between the positive and negative populations.

- [x] Tick this box to confirm that a figure exemplifying the gating strategy is provided in the Supplementary Information.
